# Differences in Mitotic Activity May Explain Observed Differences in HIF-1α Response in Cancers and Stroma

**DOI:** 10.1371/journal.pmed.0030370

**Published:** 2006-08-29

**Authors:** Khush Mittal

**Affiliations:** New York University School of Medicine, New York, New York, United States of America

I read the paper by Chi et al. with interest. The authors found a greater hypoxia response gene expression in carcinomas than stromal cells grown in vitro, but were not sure of the underlying explanation (see Discussion in [1]). The underlying explanation may be the difference in mitotic activity and hence metabolic activity between these two cell types, such that hypoxia may be present within cancer cells, although the surrounding culture media may have similar oxygen levels as the stromal cultures.

The differences in mitotic activity could also explain greater hypoxia-related gene expression in clear-cell carcinoma compared with chromophobe carcinoma, normal tissue, or oncocytoma, as clear-cell carcinomas are more active mitotically. Did the authors compare the hypoxia gene expression with mitotic activity in various tumors?
